# The Effects of Mercury on the Teeth

**Published:** 1891-01

**Authors:** E. D. Mapother


ARTICLE III.

### THE EFFECTS OF MERCURY ON THE TEETH.

BY E. D. MAPOTHER, M. D., F. R. C. S. I.

It is difficult to over-estimate the importance to dentists of questions bearing upon the prophylaxis of diseases of the teeth, so that no excuse is needful when such as that involved in the title of this paper is taken for discussion.

A large experience of the use of mercury in the treatment of syphilis, and of psoriasis and some other skin diseases, convinces me that its power of injuring the teeth has been greatly over-rated.

A hundred years ago it was given so rapidly and in such large doses, especially by quacks, that dire results were reported, and this most potent of remedies fell into undeserved disrepute. However, so recently as 1862, Professor Laycock taught: "It is a question how far the administration of mercury in early childhood or even to the parents during impregnation or growth of the ovum affects the form and development of the teeth." The male parent cannot have been meant, for no one could believe that the spermatozoon, wondrous as are its influences on the offspring, carried enough of the metal to harm the second set of teeth. From the mother the fœtus could scarcely receive, or still less could the infant store enough to interfere with the stages of dental development, the final one, that of calcification, not beginning until the sixth month in the first permanent molar.

As an example of the rapid change of scientific opinion, the following seems most noteworthy. The Government Committee on Venereal Diseases, 1864, having been instructed to inquire into "the best antidotes to injurious mercurial action," reported that any reference to the subject was almost unnecessary, and the voluminous evidence offered to it does not allude to the point.

With regard to iodiosyncrasy, I have never met a severe case of mercurial stomatitis which could be thus entirely accounted for. There had existed some discoverable cause, such as great debility, or undetected albuminuria in the general system, or some faulty condition of the teeth.

Irregularity and crowding of the teeth, giving rise to chronic gingivitis, and erosion, caries and encrustation of them, so soon encourage salivation, no matter how carefully the drug is given, that in all cases where delay is permissible, the dental surgeon should treat these conditions before a mercurial course is commenced. If severe salivation ensues from these exciting conditions, the physiological and curative effects of mercury have not been produced, yet the drug must be discontinued, and in acute diseases recovery is retarded and even loss of life may result.

My practice, therefore, not having afforded opportunities of witnessing the destructive results occasionally reported, most of the observations which follow refer to the cases of mirror-makers, whose sanitary conditions I have extensively inquired into. By them minute particles of the metal or its oxide are inhaled, and the gums and teeth suffer sooner and more surely than after administration by the skin or stomach. The edges of the gums swell and separate from the necks of the teeth, then they become spongy and hæmorrhagic. Between their everted ulcerated surfaces and the conical roots, pouches are formed, which lodge, especially in the case of the lower teeth, many products which tend to destroy the alveolo-dental membrane, the ligament of the gomphosis joint, which at the neck is especially vascular. The teeth then gradually loosen and fall out. After this, these reckless artizans follow their pernicious occupations, for the mouth gives them no further trouble. The same is recorded as to quicksilver miners.

It is very hard to produce salivation in the elderly, who are wanting in teeth, and, as all know, in the infant or young child, and the reason appears to be that the unbroken surface of the gum does not allow the access of air to, or the evolution of some gas, possibly chlorine or hydrochloric acid, from the mercurial compound. Amongst the many syphilitic infants for whom I have prescribed mercurial inunction I have never seen any stomatitis, the liver and bowels only having shown signs of its action; besides, it is undoubted that mercury may act directly on the sympathetic system and produce salivation without any local irritation. Light also appears to be promotive of such clinical change, for the effects are never so marked round the posterior teeth.

It is not fully determined in what exact form mercury circulates in the blood: that it is as a chloride, with sodium also as a base, is generally believed. By this compound, after undergoing some chemical change, the albuminoids of the tissues round the teeth would be destroyed, the vessels of the inner surface of the gums would bleed, and the cementum would die from want of vascular supply, and then blacken, as does a piece of dead bone exposed to the air. Reducing agents, such as sugar, change mercuric into mercurous salts: chlorine or hydrochloric acid would be set free, and either of these may be the destructive agent. If a ferment, which is likely to be present, were added to the latter, a digestive fluid capable of dissolving cementum would be afforded.

The whitish sticky stuff about the roots of the teeth during salivation is an albuminous substance undergoing decomposition and thereby emitting much fœtor.

It has been said that a single dose of mercury has produced toothache, but I can find no authority for the statement and no evidence whether it resulted from the drug injuring the nerve when falling into a carious cavity, or brought to it through the circulation.

Enamel, with its ninety-seven per cent. of earthy matter, cannot be susceptible of injury by mercury, and it is also proof against the attacks of bacilli, which undoubtedly can feed on the gelatin of the cementum and dentine. There is much reason to suppose that these destroyers give rise to the evolution of some acid, probably lactic, which acts on the lime salts. The indestructibility of enamel is well exemplified in the teeth of the mastodon, which have been buried for thousands of years.

There are some who believe that the loss of teeth, together with necrosis of the corresponding part of the alveolar process, which is occasionally, but rarely, seen amongst mirror-makers, is the result of merely the anæmia which mercury producces, and which firstly shows itself in the pale bloodless gum. The similar mischief, which is sometimes seen in the course of the eruptive fevers of children, has also been attributed to the poor condition of the blood, but more probably the specific poison has attacked the teeth because they are part of the dermal system. If a dose of mercury has been given, the opponents of that remedy would lay the blame on it.

It may not be out of place to mention that the French process of making mirrors by the precipitation of silver is entirely harmless, and an article at least as good and as cheap is obtained. If our workmen are still to use mercury, their factories should be provided with every possible means of allowing the particles and vapours to escape, and they should be compelled to change their clothing after work, and to take daily baths. The skin can, if urged into complete activity, eliminate much of the poison. In the many cases of mercurial stomatitis among mirror-makers, and the very few resulting from the use of the medicine, which I have seen, stoppage of the exciting cause, free exposure to air, and the frequent giving of albuminous foods, have rapidly produced amendment.

As to the local treatment of the diseased gums, it is difficult to speak with confidence until the chemical changes which occur in them shall be absolutely determined. Good effects follow the application with a very soft brush, firstly, of white of egg to neutralize the mercuric salt, and secondly, the washing of the mouth with a solution of common salt to remove it. Chlorate of potash is also a valuable remedy as a wash, in tablets slowly dissolved, or given internally. It is asserted that if used freely in the last-named way while mercury is being given, the gums never become affected.

Touching the hæmorrhagic surface of the gums with a pencil of nitrate of silver certainly checks oozing, and causes them to renew their normal hold on the neck.

To return to preventive measures, when mecury is used therapeutically, inunction or the pill form is far safer than fumigation, which really admits it, not by the unbroken cuticle, but by the mouth, much in the same way as when the injuries to mirror makers arise. This is the main point in the administration of mercury at Aix-la-Chapelle, for by ventilation of the rooms, open air exercise, ablution of the body and cleansing the mouth every two hours, the slightest salivation is avoided. Alum is there the favorite drug for local application. Although I do not believe the teeth suffer from the mercury in Stedman's powders and such nostrums, their use ought to be deprecated by every practitioner.

With regard to the mercury contained in amalgam fillings and vulcanite plates, no remarks are needed, as Sir John Tomes and a Committee of the Odontological Society in 1877 proved it to have been wholly guiltless of having caused salivation.—*London Dental Record.*

